# Effects of Corm Treatment with Cold Plasma and Electromagnetic Field on Growth and Production of Saffron Metabolites in *Crocus sativus*

**DOI:** 10.3390/ijms251910412

**Published:** 2024-09-27

**Authors:** Vida Mildažienė, Rasa Žūkienė, Laima Degutytė Fomins, Zita Naučienė, Rima Minkutė, Laurynas Jarukas, Iryna Drapak, Victoriya Georgiyants, Vitalij Novickij, Kazunori Koga, Masaharu Shiratani, Olha Mykhailenko

**Affiliations:** 1 Department of Biochemistry, Faculty of Natural Sciences, Vytautas Magnus University, Studentu Str. 10, LT-53361 Akademija, Lithuania; rasa.zukiene@vdu.lt (R.Ž.); laima.degutyte-fomins@vdu.lt (L.D.F.); zita.nauciene@vdu.lt (Z.N.); 2 Department of Clinical pharmacy, Lithuanian University of Health Sciences, A. Mickevičiaus g. 9, LT-44307 Kaunas, Lithuania; rima.minkute@kmu.lt; 3 Department of Analytical and Toxicological Chemistry, Lithuanian University of Health Sciences, A. Mickevičiaus g. 9, LT-44307 Kaunas, Lithuania; laurynas.jarukas@lsmuni.lt; 4 Department of General, Bioinorganic, Physical and Colloidal Chemistry, Danylo Halytsky Lviv National Medical University, Pekarska Str. 69, 79010 Lviv, Ukraine; iradrapak@ukr.net; 5 Department of Pharmaceutical Chemistry, National University of Pharmacy, 4-Valentinivska St., 61168 Kharkiv, Ukraine; vgeor@nuph.edu.ua; 6 Institute of High Magnetic Fields, Vilnius Gediminas Technical University, Saulėtekio al. 11, LT-10223 Vilnius, Lithuania; vitalij.novickij@vilniustech.lt; 7 Department of Immunology and Bioelectrochemistry, State Research Institute Centre for Innovative Medicine, Santariškių g. 5, LT-08406 Vilnius, Lithuania; 8 Center of Plasma Nano-interface Engineering, Kyushu University, Fukuoka 819-0395, Japan; koga@ed.kyushu-u.ac.jp (K.K.); siratani@ed.kyushu-u.ac.jp (M.S.); 9 Center for Novel Science Initiatives, National Institutes of Natural Sciences, Tokyo 105-0001, Japan; 10 Department of Pharmaceutical and Biological Chemistry, Pharmacognosy and Phytotherapy Group, UCL School of Pharmacy, 29-39 Brunswick Square, London WC1N 1AX, UK; 11 Department of Pharmaceutical Biology, Kiel University, 24118 Kiel, Germany

**Keywords:** cold plasma, crocin, electromagnetic field, HPLC, picrocrocin, safranal, saffron

## Abstract

*Crocus sativus* L. is a widely cultivated traditional plant for obtaining dried red stigmas known as “saffron,” the most expensive spice in the world. The response of *C. sativus* to pre-sowing processing of corms with cold plasma (CP, 3 and 5 min), vacuum (3 min), and electromagnetic field (EMF, 5 min) was assessed to verify how such treatments affect plant performance and the quality and yield of herbal raw materials. The results show that applied physical stressors did not affect the viability of corms but caused stressor-dependent changes in the kinetics of sprouting, growth parameters, leaf trichome density, and secondary metabolite content in stigmas. The effect of CP treatment on plant growth and metabolite content was negative, but all stressors significantly (by 42–74%) increased the number of leaf trichomes. CP3 treatment significantly decreased the length and dry weight of flowers by 43% and 60%, respectively, while EMF treatment increased the length of flowers by 27%. However, longer CP treatment (5 min) delayed germination. Vacuum treatment improved the uniformity of germination by 28% but caused smaller changes in the content of stigma compounds compared with CP and EMF. Twenty-six compounds were identified in total in *Crocus* stigma samples by the HPLC-DAD method, including 23 crocins, rutin, picrocrocin, and safranal. Processing of *Crocus* corms with EMF showed the greatest efficiency in increasing the production of secondary metabolites in saffron. EMF increased the content of marker compounds in stigmas (crocin 4: from 8.95 to 431.17 mg/g; crocin 3: from 6.27 to 164.86 mg/g; picrocrocin: from 0.4 to 1.0 mg/g), although the observed effects on growth were neutral or slightly positive. The obtained findings indicate that treatment of *C. sativus* corms with EMF has the potential application for increasing the quality of saffron by enhancing the amounts of biologically active compounds.

## 1. Introduction

Pre-sowing treatment of seeds or other planting material with physical stressors is a modern method of plant growth stimulation via triggering numerous physiological and biochemical changes, including an increase in the content of valuable secondary metabolites [1,2,3,4,5]. The application of physical stressors in pre-sowing treatment to corm plants, including *Crocus sativus*, has not been studied before.

The dried red stigmas of *Crocus sativus* L. (Iridaceae) are used as saffron, one of the oldest and most expensive spices in the world, due to the laborious way it is harvested and processed. Saffron is used in Mediterranean and Asian cuisines to improve food taste, flavor, and color and is cultivated in various parts of the world, mostly in Iran, India, Spain, Greece, and Turkey [6]. The growing interest in cultivating and using saffron in Ukraine [7] and different EU countries may increase agricultural production revenues.

Saffron gains its special value due to its chemical composition. It contains significant amounts of unique compounds: apocarotenoids, crocins, as well as picrocrocin and safranal, which are formed by zeaxanthin cleavage followed by specific glycosylation steps [8]. Crocin determines the color of saffron, picrocrocin provides a bitter taste, and safranal gives a specific aroma [8]. Besides apocarotenoids, a wide variety of other biologically active compounds have been isolated from *Crocus* stigmas. More than 150 components, including lipophilic and hydrophilic carbohydrates, proteins, amino acids, minerals, mucilage, starch, gums, vitamins (such as riboflavin and thiamine), pigments, alkaloids, and saponins, have been found in different parts of *C. sativus* [9]. Due to the biological activity of secondary metabolites, saffron extracts have numerous therapeutically relevant effects, including anticatarrhal, anticancer, anti-inflammatory, antimicrobial, antioxidant, laxative, eupeptic, antispasmodic, antidepressant, respiratory decongestant, nerve sedative, stomachic, expectorant, carminative, diaphoretic, anodyne, gingival sedative, galactogogue, and effects against amenorrhea, dysmenorrhea, and others [9]. Therefore, the growing demand for saffron production is driven by pharmacological applications [7].

The typical low yield of saffron is attributed partly to primitive agronomic practices. The quality of saffron is related to the concentration of secondary metabolites and determined according to the international ISO 3632 standards [10], the European Medicines Agency, Food and Drug Administration, and European Pharmacopeia on the international trading market. Saffron yield and quality are influenced by cultivation methods and the environment [11]. It also depends on the methods used for its processing, such as drying, extraction, separation, storage, and quantification stages [12]. The traditional ways for increasing productivity through breeding are restrained because *C. sativus* is a perennial sterile plant characterized by vegetative reproduction using corms. Several attempts to develop superior varieties of saffron via inducing genetic variability have been carried out using tissue culture and hybridization [13,14]. However, propagation through corms offers none or very few genetic variations. Considering the high price and low production yield of valuable saffron components, innovative technologies to increase the production of saffron marker compounds are in high demand. This study aimed to test the hypothesis that pre-sowing corm processing with physical stressors induces stress, leading to increased amounts of secondary metabolites in saffron. The idea is based on similar effects observed in medicinal plants after seed treatment with cold plasma (CP) or electromagnetic field (EMF).

It has been recognized recently that pre-sowing seed treatment with CP or EMF may result in stimulation of plant growth as well as in numerous physiological and biochemical changes, including an increase in the amounts of valuable secondary metabolites [1,2,3,4,5]. For example, irradiation of *Echinacea purpurea* seeds with low-pressure CP and radiofrequency (RF) EMF induced a strong increase in the content of vitamin C and phenolic acids in the leaves of seedlings 3 months after sowing [15]. Changes in the secondary metabolite amounts after seed treatments with CP and EMF were also observed in red clover [16,17], common buckwheat [18], and other plants [13]. CP has been applied for seed treatment in a wide variety of plant species (see reviews [10,11,12,13]), but only one study [19] has reported results on planting material of geophyte plants [20], which are propagated through underground storage organs such as bulbs, tubers, corms, or rhizomes. In several recent studies, CP was used to irradiate dried saffron, revealing its potential for microbial decontamination and for increasing color intensity while maintaining the primary quality properties of saffron [20,21,22].

As an extension of previous studies and taking into account the important pharmacological properties of saffron secondary metabolites, this experiment studied the effect of corm treatment with several physical stressors on plant germination parameters and the composition of saffron metabolites.

## 2. Results

### 2.1. Effects on Sprouting Kinetics, Seedling Growth, and Number of Leaf Trichomes

**Figure 1:**
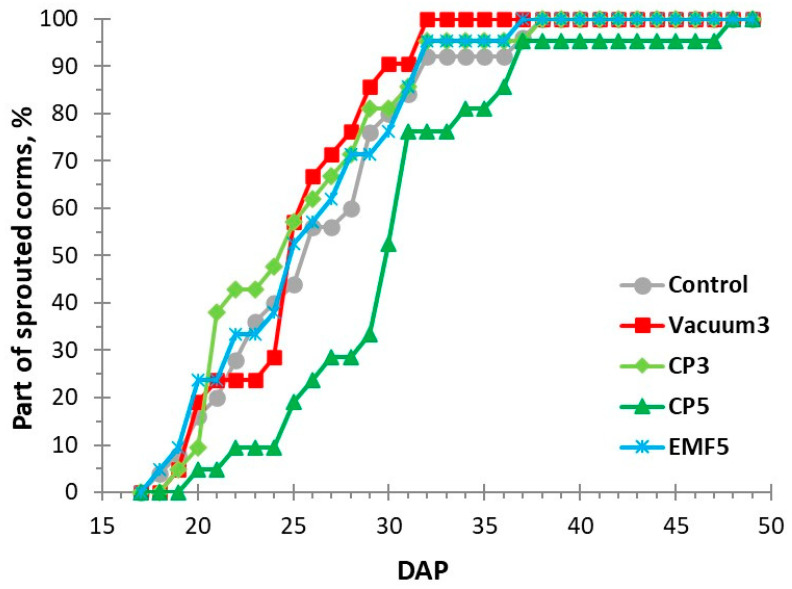
Kinetic curves of *Crocus sativus* corm sprouting following treatments with physical stressors: cold plasma for 3 and 5 min (CP3 and CP5, respectively), vacuum for 3 min (Vacuum3), and electromagnetic field for 5 min (EMF5), along with untreated controls. DAP—days after planting.

The control and treated corms of *C. sativus* were planted in field experimental plots 4 days after treatment. Corms started sprouting on the 17th day after planting (DAP), and their sprouting dynamics were observed and registered every other day until the 49th DAP (Figure 1).

Richards plots [15,23] were used to quantify the main indices of sprouting kinetics, and the results are presented in Table 1. Treatments did not affect the maximal sprouting percentage, indicating that corms were equally viable in all groups. CP5 treatment reduced sprouting (increased the median sprouting half-time) by 15% so that 50% of the corms sprouted with a 4-day delay compared with the control. The uniformity of corm sprouting was increased in the Vacuum3-treated group, as indicated by a 28% decrease in Qu compared with the control.

**Figure 2:**
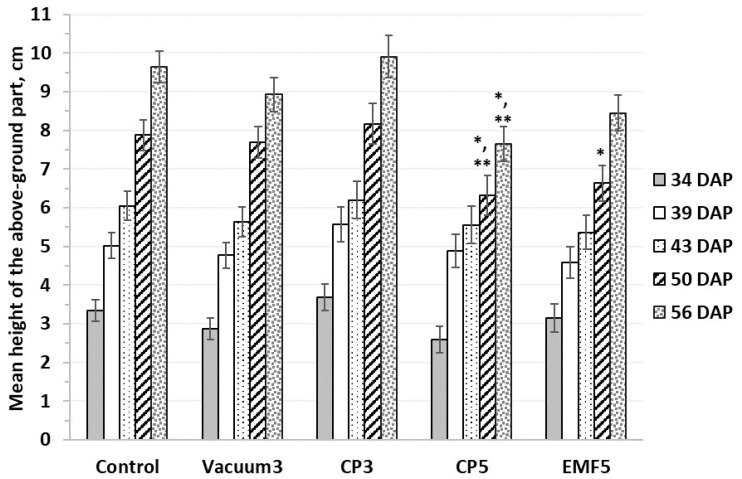
The dynamics of seedling growth estimated by sprout height. Mean values ± standard error are presented (n = 24); *, significantly different from the control group (*p* < 0.05); **, significantly different from the Vacuum3 group (*p* < 0.05). DAP—days after planting.

The height of the aerial part of the seedling did not differ among groups in the early stages of sprout growth (Figure 2). Seedling growth in the period of 56 DAP was similar in the Vacuum3, CP3, and EMF5 groups compared with the control, except for a 15% reduction in height observed in the EMF5 seedlings on 50th DAP. However, the CP5 group showed a significant reduction in seedling height during the 50–56 DAP interval, with 19% and 28% smaller heights, respectively, compared with the control, and 17% and 13% smaller, respectively, compared with the Vacuum3 group.

The relative number of sprouted buds per corm in the experimental groups is presented in Table 2. A larger variation and an increased number of buds per corm were observed in the Vacuum3 group, while the bud profile in the EMF5 corms was the most similar to the control (with prevailing three buds per corm). The largest proportion of corms with two buds per corm was found in the CP-treated groups, with a stronger shift to a smaller number of buds observed after the CP5 treatment.

**Figure 3:**
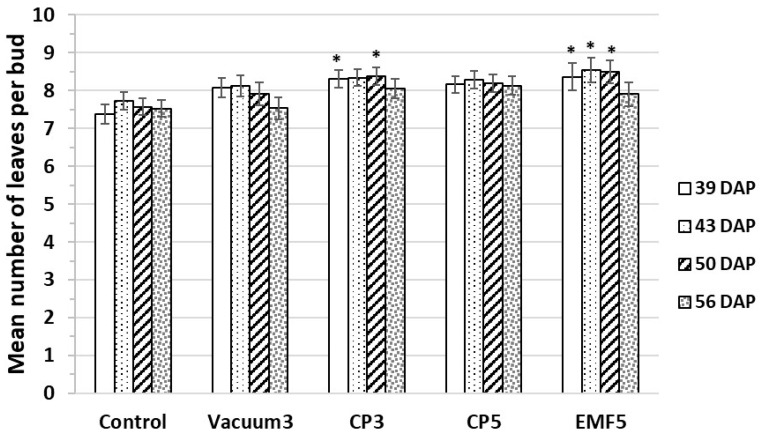
The mean number of leaves per bud in seedlings. Mean values ± standard error are presented (n = 24); *, significantly different from the control group (*p* < 0.05). DAP—days after planting.

The mean number of leaves developed per bud was counted starting from the 34th DAP till the 56th DAP (Figure 3). The number of leaves in the Vacuum3 and CP5 groups was not different from the control, while CP3 and EMF5 treatments increased the mean number of leaves per bud by approximately 10%.

**Figure 4:**
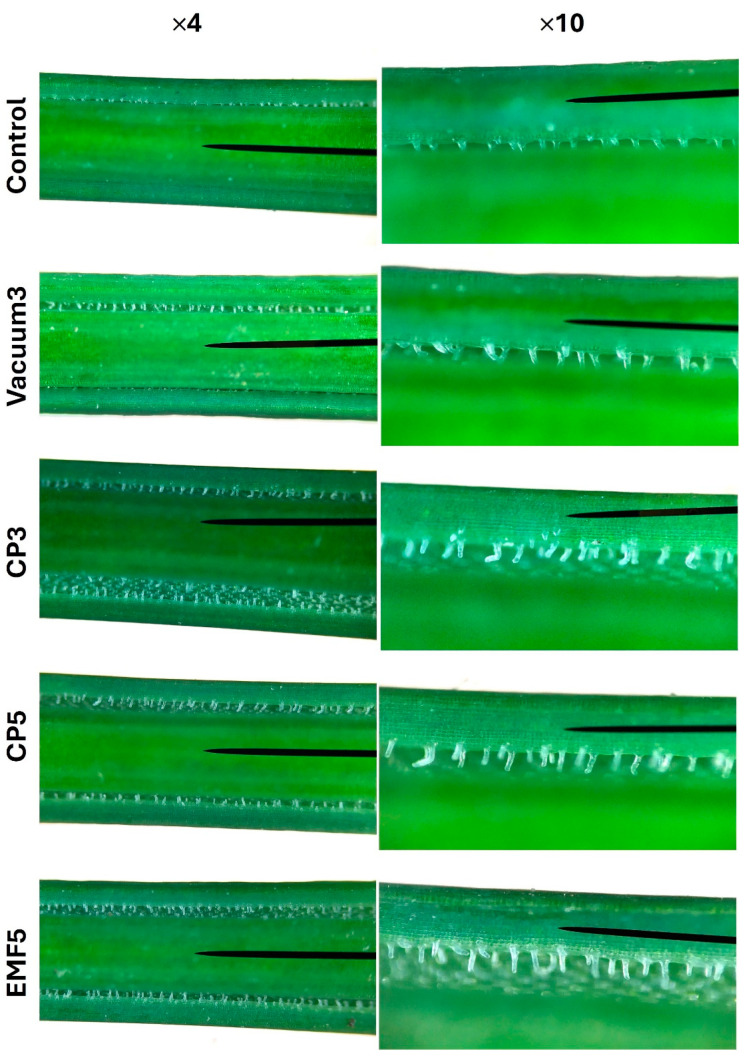
Trichomes in *C. sativus* leaves on the abaxial surface of crypt aperture (the position at the distance between 8 and 9 cm from the tip of a leaf was used for the image). ×4, ×10—magnification four and ten times, respectively.

**Figure 5:**
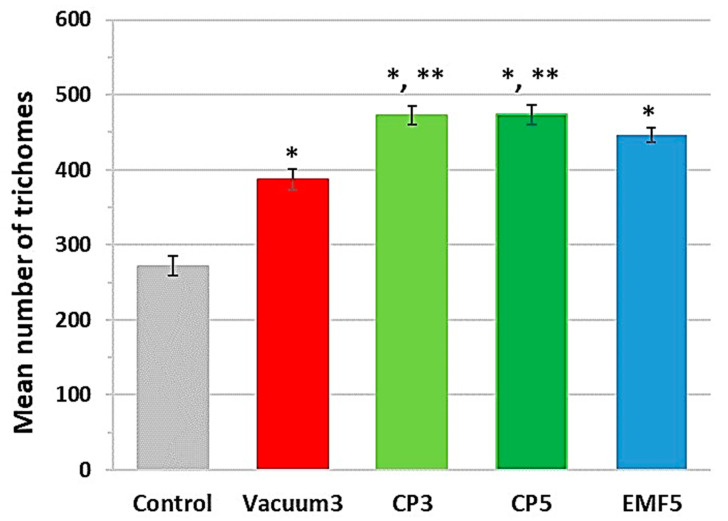
The number of trichomes in a 1 cm long section located between 8 and 9 cm from the tip of the leaf. Mean values ± standard error are presented (n = 24); *, significantly different from the control group (*p* < 0.05); **, significantly different from the Vacuum3 group (*p* < 0.05).

Microscopic analysis of leaf trichomes [23,24] was performed on 56th DAP corms. Simple unicellular trichomes of different sizes were found on the abaxial surface of the crypts (Figure 4). Treatments of *C. sativus* corms with all stressors resulted in an increased density of leaf trichomes (Figure 5). The largest effect (74%) was observed in the CP-treated groups (CP3 and CP5), while Vacuum3 and EMF5 treatments were slightly less effective (43 and 63%, respectively). The density of trichomes in both of the CP-treated groups was 23% higher than in the Vacuum3 group. In addition, the size of trichomes was larger in all treated groups compared with the control, as seen in Figure 4.

Flowers were collected from November 6th until December 3rd, the day the experiment was concluded due to winter cold. CP5 group did not flower at all during the first year after planting corms. Morphometric parameters of flowers collected from the other treatment groups and the control are presented in Table 3.

The results demonstrate that corm processing with CP3 significantly reduced the length and dry weight (43 and 60%, respectively) of the flowers. This indicates a negative effect of CP on flowering, which intensified with the duration of treatment. Consequently, plants from the CP5 group did not develop flowers. EMF5 treatment had a positive effect on flowering and increased flower length by 27% compared with the control. No statistically significant differences between experimental groups in the morphometric parameters of the stigmas were observed.

### 2.2. Effects on Amounts of Secondary Metabolites in the Stigma of C. sativus

**Figure 6:**
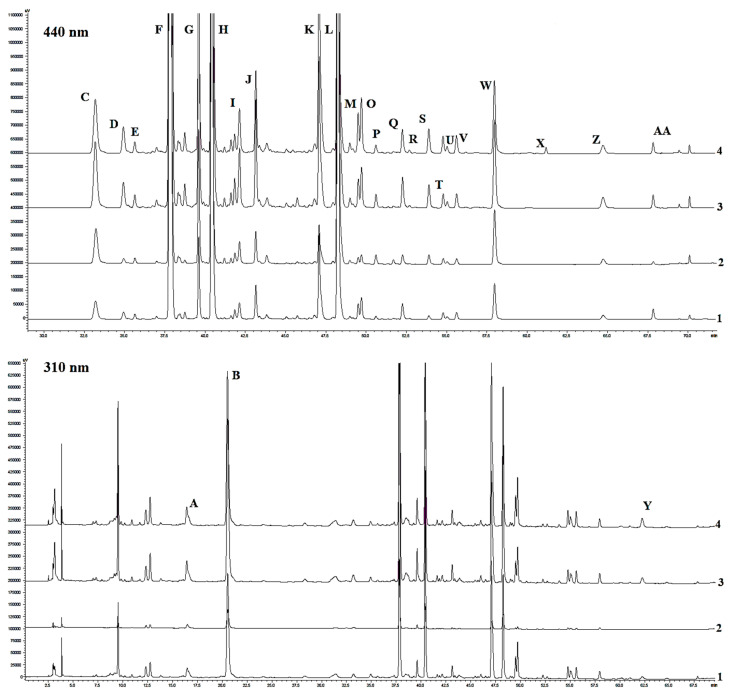
HPLC chromatogram of *C. sativus* stigma methanol extracts. Detection wavelength—440 nm and 310 nm.

The amounts of secondary metabolites in dried stigma extracts were quantified by HPLC analysis [25,26]. The typical chromatogram is shown in Figure 6, and the chromatographic characteristics of the identified esters: crocetin, safranal, picrocrocin, and rutin, their retention time (t*_R_*) at 440 nm, and UV-visible spectra are presented in Table 4. In total, 26 compound peaks were identified in the HPLC chromatograms of *Crocus* stigma extracts. However, six compounds were not identified. Of the 17 different derivatives of crocetin, 15 compounds have been identified. The content of pricrocrocin, rutin, and safranal was estimated.

**Figure 7:**
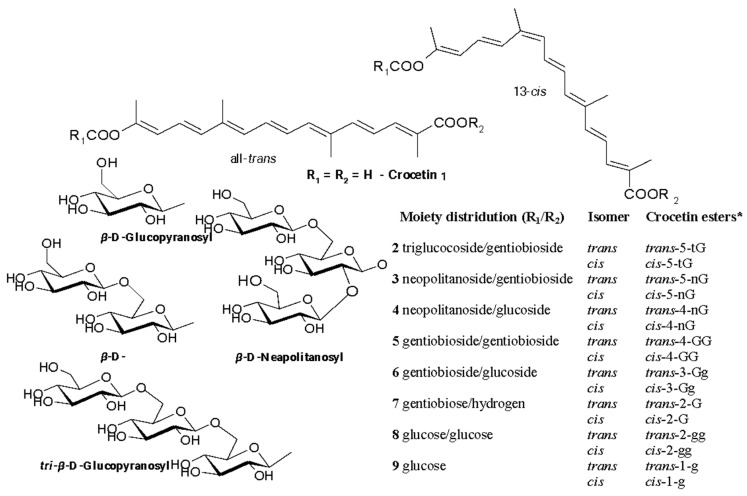
Structural formulas of esters of *trans*- and *cis*-crocetin and substituents at R1 and R2. Nomenclature according to [27]. Note: * Meaning of each letter in the abbreviation of the name of each crocetin ester: Number (5, 4, 3, 2)—number of glucose molecules attached to the molecule of crocetin; t—triglucose; G—gentiobiose; g—glucose; n—neapolitanose.

The compounds of the crocetin group dominated stigma extracts in the control, with *trans*-crocetin di(*β*-D-gentiobiosyl) ester, *trans*-crocetin mono(*β*-D-gentiobiosyl) ester, *cis*-crocetin di(*β*-D-gentiobiosyl) ester, and *cis*-crocetin (*β*-D-gentiobiosyl)(*β*-D-glucosyl) ester present in amounts as high as 114.3 mg/g, 49.0 mg/g, 25.4 mg/g, and 23.9 mg/g of dry weight, respectively (Table 5). The chemical structures of crocetin derivatives are presented in Figure 7.

**Figure 8:**
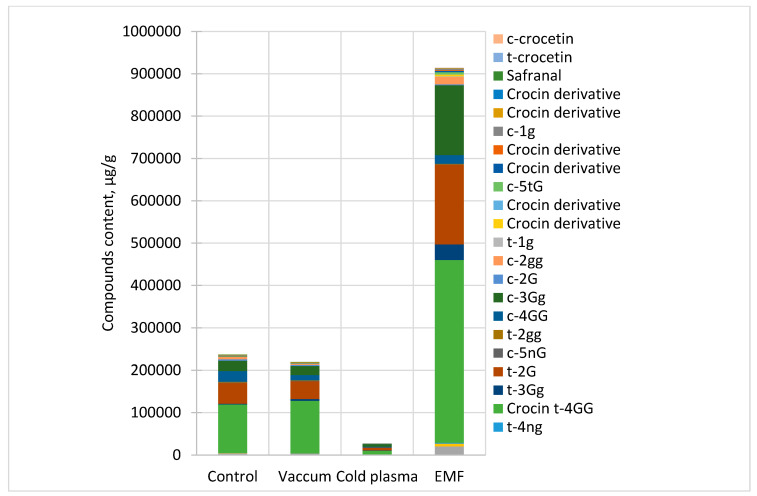
Distribution of the compounds in *C. sativus* stigma depending on the pre-sowing treatment method.

The results showed that treatment of corms with CP, vacuum, and EMF induced significant changes in the content of secondary metabolites in *Crocus* stigma, and the change strongly depended on the nature of the stressor (Figure 8). The changes induced by Vacuum3 treatment were smaller compared with those induced by CP3 and EMF5. Vacuum3 had no effect on the amount of certain metabolites (e.g., B, C, D, F, etc.), increased the amount of some (A, G, S), and decreased the amount of other metabolites (H-O, Q, V, W). CP3 treatment had a strong negative effect on the content of secondary metabolites. It significantly reduced the amount of all metabolites, including more than 10 times of some of them (e.g., F, L, M.O). On the contrary, EML5 had a significant positive effect on the accumulation of many secondary metabolites in *Crocus* stigma. For example, EMF5 increased the amount of G and S twenty-two and nineteen times, C and L nine and seven times, and F and H four times. On the other hand, EMF5 did not change the amount of rutin and reduced the amounts of I, J, K, and M metabolites in the stigma. The amount of picrocrocin was increased by the exposure to Vacuum3 and EMF (34 and 18%) and strongly reduced (45%) by CP3. Vacuum3 did not change the amount of safranal, while CP3 reduced it five times, in contrast to EML5, which induced a two-fold increase. Certain compounds that were not present in the control group (e.g., P, U, X, AA) were only identified in the stigma after EMF5 treatment, while R and Z synthesis was induced by both CP3 and EMF5, with a stronger effect observed with the latter treatment.

## 3. Discussion

The experimental study on the effects of corm treatment with CP, vacuum, and EMF on sprouting, plant growth, and secondary metabolite content in the stigma was performed to estimate the potential of such treatments to improve saffron production yield and quality. Our hypothesis was based on numerous reports about the positive effects of pre-sowing seed treatment on the growth and metabolite content of medicinal plants, including those used as herbal supplements, such as purple coneflower [15], red clover [16,17], industrial hemp [28], and Norway spruce [29]. However, similar research on the planting material of geophyte plants is limited by a single report [19]. In the said study, low-pressure radio frequency oxygen plasma was applied for 60 s on peeled garlic cloves of a spring-planted Slovenian autochthonous cultivar “Ptujski spomladanski”. This resulted in increased water uptake during germination as well as early seedling growth stimulation under laboratory conditions. However, treatments did not have an effect on garlic yield after 4 months of cultivation in a field experiment. The effects of treatments on the amounts of secondary metabolites in garlic tissues were not assessed in this study [19]. The results of our study revealed that a strong impact on secondary metabolite content may not be associated with the obvious effects on plant seedling growth.

Increased generation of the protective secondary metabolites is a common hallmark of plant stress response controlled by a complex network of regulatory molecules, including phytohormones [30]. The main phytohormones responsible for stress-induced enhancement of secondary metabolite synthesis are salicylic acid and jasmonates [31]. Synthesis of carotenoids (including crocins) in plants is also under the tight control of phytohormone signaling [32,33]. It was experimentally demonstrated that treatment with cold plasma and electromagnetic field induces a rapid shift in the balance of phytohormones in dry seeds [34,35,36,37,38]. According to the suggested hypothesis [4], seed treatment-induced changes in seed phytohormone composition are further retained in growing plants, entailing the modified patterns in phytohormonal networks and generating a multitude of changes in gene expression, resulting in numerous effects on plant biochemical and physiological processes, including the activity of biosynthetic pathways of secondary metabolites. Such explanation is based on the reported examples of enhanced activity of the enzymes in the secondary metabolite biosynthesis pathways induced by seed treatment with CP in hemp [39], pink periwinkle [40], and blue sage [41], as well as activity of two key enzymes of the pathway of carotenoid biosynthesis, phytoene-synthase and phytoene desaturase, in bitter melon [42]. The results of our study show that processing of *C. sativus* corms with CP has only an adverse impact on the biosynthesis of crocins, in contrast to strong positive effects elicited by EMF treatment. We suggest that changes in the balance of phytohormones and signal transduction pathways in corms and growing seedlings are dependent on the nature of the stressor.

The main reason why CP (or vacuum) has been widely applied for seed treatment but not for planting material of geophytes is the much smaller size and low water content of orthodox seeds, which undergo desiccation during maturation and the moisture content in the anhydrobiotic state decreases to less than 10 percent [43,44]. The anhydrobiotic state is crucially important for seed adaptability to environmental changes, stress resistance, and longevity. In contrast, moisture content in the planting material of geophytes is much higher; for example, 72–74% of water was detected in *C. sativus* corms [45]. Numerous chemical products are formed during the interaction of aggressive particles from the CP gaseous phase with water in the plasma target interphase, giving rise to damaging factors such as nitric acids or a rapid pH drop [46]. In addition, water evaporates rapidly under high vacuum conditions, and that can cause a structural disruption and collapse of biological structures [47]. Assumptions about the potential damage have possibly held researchers back from testing the effects of CP (or vacuum) on botanical samples other than anhydrobiotic seeds. In this respect, EMF seems to be a more acceptable stressor for corms or bulbs containing relatively high amounts of moisture.

Nevertheless, the results obtained in this study provide evidence that the short duration of CP or vacuum treatment did not reduce the viability of *C. sativus* corms, although CP did not stimulate sprouting like it was reported earlier for garlic cloves [19]. However, numerous negative effects of CP treatment were observed in the later stages of seedling development, including reduced height, percentage of sprouted buds per corm, length, and number of developed flowers (adverse effects were much stronger in the CP5 group). Vacuum3 treatment did not have obvious effects on plant growth but increased uniformity of sprouting and the number of sprouted buds per corm, possibly indicating stimulation of secondary buds. EMF treatment exerted no negative effects, did not change corm sprouting kinetics or plant height, but increased the mean number of leaves per bud and the length of flowers, which was associated with a strong positive effect of EMF on the content of secondary metabolites in the dried stigma of *C. sativus*. At the same time, the adverse effects of the CP treatment were related to a drastic decrease in the secondary metabolite content. The effects of Vacuum3 on secondary metabolite content were less pronounced; the concentrations of certain compounds were increased, while the amounts of other metabolites were decreased.

Interestingly, despite the different effects of CP, vacuum, and EMF on plant growth and secondary metabolites in stigma, corm treatment with all stressors strongly increased the density and size of leaf trichomes. Trichomes, the outgrowths of epidermal cells present on the aerial parts of most plant tissues, have an enormous variety in terms of morphology and perform a range of protective and other functions [24]. One of the main functions of trichomes is the synthesis and accumulation of secondary metabolites and defensive proteins [24], but the impact of the different types of stress on amounts of trichomes is poorly understood [48,49]. Our results support the hypothesis that corm exposure to abiotic stress leads to an increase in the density of leaf trichomes, although these effects do not correlate with the changes in secondary metabolite amounts in dried stigma.

At least two studies have been published on the application of cold plasma technology on *Crocus* stigmas to increase the content of key compounds, such as crocin and safranal [50,51]. Unfortunately, a decrease in marker compounds, namely crocin esters and safranal, was observed after the treatments, and no trans-2G, cis-4GG, or cis-3Gg compounds were observed at all in the samples after cold plasma treatment with Ar/10% O2 at 12 kV [52]. However, these treatments were applied to the stigma itself, not to the corms. Cold plasma technology generates reactive species that can interact with plant tissues, potentially enhancing the production of secondary metabolites at the plant germination level.

In summary, our study demonstrated that corm treatment with CP, vacuum, and EMF does not have an effect on corm viability, and the effects on secondary metabolite amount in stigma are much stronger compared with the effects observed on plant growth and development. The most interesting finding was the obvious potential of corm treatment with EMF to increase the amounts of valuable biologically active compounds in saffron.

## 4. Materials and Methods

### 4.1. Plant Material and Seedling Cultivation

Corms of *Crocus sativus* L. were obtained from a Ukrainian manufacturer (“Shafran Lyubimovske” farm, Lyubimovka village, Kherson region, Ukraine). Raw material was collected and identified by Dr. Mykhailenko, and samples were deposited at the Herbarium of Botany Department of the National University of Pharmacy, Kharkiv, Ukraine (voucher specimen No. 20198).

The field experiment was carried out in the experimental plots at Vytautas Magnus University Botanical Garden (Kaunas, Lithuania, coordinates 54.868567, 23.911813). Four days before planting, corms were treated with physical stressors: low-pressure cold plasma for 3 and 5 min, further denoted as CP3 and CP5; vacuum for 3 min (as an additional control for CP), denoted as Vacuum3; and electromagnetic field for 5 min, denoted as EMF5. The control and treated corms (24 corms per treatment group) were planted in half-shaded, well-cultivated loam soil in 3 rows (replicates) at the beginning of autumn in 2020. Corms were planted at a depth of 10 cm, keeping a distance of 20 cm between corms, 20 cm between rows of the same treatment group, and 45 cm spacing between different treatment groups. The experimental plots were irrigated manually two times in the first two weeks after planting to reduce the impact of summer drought. No fertilization was applied.

### 4.2. Corm Treatment by CP, Vacuum, and EMF

Corms were irradiated with CP using a low-pressure not-thermal plasma device constructed by Prof. Koga and Prof. M. Shiratani (Kyushu University, Japan). The hermetic chamber (length 152 mm, diameter 102 mm) of the CP generator was made of stainless steel (SUS-304). To generate the discharge, a helical power electrode-antenna (20 mm long and 15 mm in diameter) installed at the top of the chamber was switched to a high-frequency voltage of 430 MHz by a radio transmitter (the power supply was 45 W). The reactor chamber performed the function of the other electrode. Three corms were placed at the bottom of the chamber per treatment. The distance between the upper electrode and the corms adjusted with additional glass stands was 8 cm. Atmospheric air was used as a gaseous phase, and a vacuum (100 Pa) was created before the discharge by pumping out air with a pump system. The same chamber was used to treat the corms with a vacuum (100 Pa) for 3 min as an additional control for the CP treatment.

Corms were treated with radiofrequency (RF) EMF using a pulsed magnetic field generator (peak parameters 100 kHz, 0–10 mT) designed at Vilnius Gediminas Technical University by Prof. Vitalij Novickij. The treatment was performed for 5 min (the treatment is further abbreviated as EMF5) using 100 kHz, 400 ± 50 μT oscillating magnetic field at atmospheric pressure and room temperature. Three corms were treated at one time, keeping them at a 3 cm distance above the center of the induction coil.

### 4.3. Analysis of Sprouting and Plant Morphometric Parameters

Sprouting dynamics of *Crocus* corms were observed and registered every other day for 32 days after the first sprout appeared. Richards plots were constructed [15,23] to quantify the main indices of plant sprouting kinetics: Vi (%)—the maximal sprouting percentage indicating corm viability, Me (days)—the median sprouting time (t_50%_) indicating halftime of corm sprouting rate, Qu (days)—the quartile deviation indicating the dispersion of sprouting time or sprouting uniformity.

Morphometric parameters were measured two weeks after the first sprout appeared. The height of the above-ground plant part was measured, and the mean number of sprouted buds per corm, as well as the mean number of leaves per bud, were counted starting from the 34th till the 56th DAP.

Trichomes were counted on the abaxial side of a leaf to estimate the possible response of growing seedlings to stress induced by corm treatments. A light microscope was used to quantify trichomes, and the size of the microscopic view was calibrated using a Motic calibration slide. Simple unicellular trichomes on the crypt aperture were counted on both edges of the crypt at 1 cm, approximately in the middle of a leaf (in the distance between the 8th and the 9th centimeters from the tip of a mature leaf).

*C. sativus* flowers were collected manually, and after morphometric measurements, their stigmas were separated and dried for 2–3 h at 50 °C under forced air. Dried stigmas were stored in dark glass jars at 4 °C.

### 4.4. Sample Preparation and HPLC-DAD Analysis

Saffron samples (0.1 g each) were ground and extracted with methanol/water (85:15 *v*/*v*, 10 mL). The extractions were performed in an ultrasonic bath (Wise Clean WUC-A06H, Witeg Labortechnik GmbH, Wertheim, Germany) with a working frequency of 33 kHz. The samples were sonicated at room temperature for 30 min, and the resulting solutions were filtered through a membrane filter (0.45 μm) and analyzed.

Acetonitrile and methanol of HPLC grade were purchased from Roth GmbH (Karlsruhe, Germany), while water was purified through a Milli-Q system (Millipore, Bedford, MA, USA). Standards compounds crocin (CAS number 42553-65-1) and safranal (CAS number 116-26-7) with a purity of >90% were purchased from Sigma-Aldrich (St. Louis, MO, USA).

Crocin, safranal, and picrocrocin were identified and quantified using a Shimadzu Nexera X2 LC-30AD high-performance liquid chromatography system (HPLC, Shimadzu, Kyoto, Japan) composed of a quaternary pump, an online degasser, a column temperature controller, the SIL-30AC autosampler (Shimadzu, Japan), the CTO-20AC thermostat (Shimadzu, Japan), and SPD-M20A diode array detector (DAD). All data processing was carried out using LabSolutions Analysis Data System (Shimadzu Corporation) as described earlier [38]. Chromatographic separation was performed with an ACE C18 column (250 mm × 4.6 mm, 5.0 μm; Radnor, PA, USA). Elution was performed at a flow rate of 1 mL/min. The binary solvent system of the mobile phase consisted of solvent A (0.1% acetic acid in water) and solvent B (acetonitrile). All solvents were filtered through a 0.22 μm membrane filter after ultrasonic degassing. Following that, a linear elution gradient was applied: 0–8 min, 5–15% B; 8–30 min, 15–20% B; 30–48 min, 20–40% B; 48–58 min, 40–50% B; 58–65 min, 50% B; 65–66 min, 50–95% B. Column temperature was constant at 25 °C. The injection volume of the sample solution was 10 μL. The DAD detector was set at 260, 310, and 440 nm wavelengths for picrocrocin, safranal, and crocin, respectively. Standard solutions, including crocin and safranal, were used for calibration of a standard curve using an external standard method. Analyses were performed in duplicate. Identifications of crocin were carried out using the UV–Vis spectrum, the retention time of the crocin standard at 440 nm according to the HPLC-DAD method. Picrocrocin content in the extracts was recalculated as a safranal equivalent. Safranal identification was carried out using the UV–Vis spectrum and the retention time of the safranal standard at 310 nm according to the HPLC-DAD. The calibration curve for safranal and crocin was constructed by plotting the chromatogram peak area at absorption maxima versus the known concentration of the standard solution.

### 4.5. Statistical Analysis

Means of various parameters between the control and treatment groups were compared using Student’s *t*-tests for independent samples. Differences were considered to be statistically significant at *p* ≤ 0.05. The number of measured plants in the control and treatment groups was 24 (analysis of morphometric parameters), or 8 plants for one replicate. Three extract samples were analyzed via the HPLC-DAD method. Data are presented as means of 3 independent repetitions ± standard error of the mean.

## 5. Conclusions

Treatment of *Crocus sativus* corms with CP, vacuum, and EMF did not reduce their viability; the final sprouting in all groups was 100% under field experiment conditions. However, the longer duration of the CP treatment (5 min) reduced sprouting, while more uniform sprouting of corms was observed in the vacuum-treated group.

The effects of three different stressors in the later stages of plant growth strongly depend on the nature of the stressor. The adverse effects of the CP treatment on plant height, number of sprouted buds, length, and number of flowers increased with the treatment duration. The vacuum had a minor positive impact on sprouting, while EMF treatment had a positive effect, e.g., increased the number of *Crocus* leaves per bud and the length of flowers. Treatment with all stressors significantly increased the density of leaf trichomes.

Corm processing with EMF presents a promising application for enhancing saffron production, improving the quality, and increasing the content of its bioactive compounds. The obtained results indicate that corm exposure to EMF can stimulate growth, increase biomass, and improve the yield of saffron stigmas, which are the primary source of valuable compounds like crocin, picrocrocin, and safranal. Additionally, EMF treatments may enhance the synthesis and accumulation of these key metabolites, leading to higher-quality saffron with greater medicinal and culinary value. Overall, integrating EMF treatment into saffron cultivation could be a sustainable and innovative approach to boosting both yield and quality, providing significant economic benefits to saffron producers. However, the reproducibility and persistence of this effect have to be studied further to optimize corm treatment conditions.

## Figures and Tables

**Table 1:** Indices of *C. sativus* sprouting kinetics after planting in field plots.

Indice/Treatment Group	Control	Vacuum3	CP3	CP5	EMF5
Vi (%)	100.0 ± 0.0	100.0 ± 0.0	100.0 ± 0.0	100.0 ± 0.0	100.0 ± 0.0
Me (days)	25.6 ± 0.5	25.1 ± 0.4	24.5 ± 1.1	29.5 ± 0.5 *	25.3 ± 1.1
Qu (days)	3.6 ± 0.3	2.6 ± 0.1 *	3.3 ± 0.2	3.0 ± 0.1	3.2 ± 0.4

Vi, the maximal sprouting percentage; Me, the median sprouting half-time; Qu, the quartile deviation. Mean values ± standard error are presented (8 corms used in each of three replicates, n = 24 corms in one group); *, significantly different from the control group (*p* < 0.05).

**Table 2:** The percentage of sprouted buds per corm at the 56th DAP.

Bud Number per Corm	Control	Vacuum3	CP3	CP5	EMF5
1	16.0	9.5	14.3	33.3	9.5
2	12.0	23.8	38.1	33.3	28.6
3	36.0	19.0	23.8	28.6	38.1
4	28.0	28.6	14.3	4.8	19.0
5	4.0	9.5	9.5	0.0	4.8
6	4.0	4.8	0.0	0.0	0.0
7	0.0	4.8	0.0	0.0	0.0

Results are presented as percent of corms with the indicated number of buds (n = 24 corms).

**Table 3:** The morphometric parameters of the collected flowers.

Morphometric Parameter	Control (6)	Vacuum3 (6)	CP3 (4)	EMF5 (7)
Flower length, cm	7.90 ± 0.00	8.00 ± 0.50	4.50 ± 0.50 *	10.00 ± 0.00 *
Flower fresh weight, g	0.43 ± 0.05	0.46 ± 0.05	0.22 ± 0.03	0.52 ± 0.07
Flower dry weight, g	0.05 ± 0.002	0.05 ± 0.003	0.02 ± 0.001 *	0.07 ± 0.01
Pistil length, cm	4.10 ± 0.20	4.07 ± 0.23	3.80 ± 0.18	3.87 ± 0.19
Pistil fresh weight, g	0.03 ± 0.003	0.03 ± 0.003	0.02 ± 0.002	0.03 ± 0.009
Pistil dry weight, g	0.004 ± 0.0005	0.007 ± 0.002	0.004 ± 0.0007	0.004 ± 0.0003

Mean values ± standard error are presented (the number of flowers indicated in parenthesis); *, significantly different from the control group (*p* < 0.05).

**Table 4:** Peak number, retention time (t*_R_*), and content of each chromatographic peak corresponding to crocetin esters (crocins) tentative identification * in *C. sativus* stigma extract.

Compound	No Glucose Moieties	Abbreviation **	t*_R_* (min)	UV/Vis, λmax, nm	MW, g·mol^−1^	MolFor
*trans*-crocetin (tri-*β*-D-glucosyl)(*β*-D-gentiobiosyl) ester	Crocin **5**	t-5tG	33.190	262, 332sh, 443, 468	1138	C_50_H_74_O_29_
*trans*-crocetin (*β*-D-neapolitanosyl)(*β*-D-gentiobiosyl) ester	-	t-5nG	34.928	234, 300sh, 418, 439	1138	C_50_H_74_O_29_
*trans*-crocetin (*β*-D-neapolitanosyl)(*β*-D-glucosyl) ester	-	t-4ng	35.630	263, 441, 466	976	C_44_H_64_O_24_
*trans*-crocetin di(*β*-D-gentiobiosyl)ester	Crocin **4**	t-4GG	37.825	261, 331sh, 440, 466	976	C_44_H_64_O_24_
*trans*-crocetin (*β*-D-gentiobiosyl)(*β*-D-glucosyl) ester	Crocin **3**	t-3Gg	39.608	261, 327sh, 440, 466	814	C_38_H_54_O_19_
*trans*-crocetin mono(*β*-D-gentiobiosyl) ester	Crocin **2**	t-2G	40.424	261, 331sh, 440, 466	652	C_33_H_44_O_14_
*cis*-crocetin (*β*-D-neapolitanosyl)(*β*-D-gentiobiosyl) ester	-	c-5nG	42.119	242, 260, 414, 436	1139	C_50_H_74_O_29_
*trans*-crocetin di(*β*-D-glucosyl) ester	Crocin **2′**	t-2gg	43.147	261, 322, 439, 465	652	C_33_H_44_O_14_
*cis*-crocetin di(*β*-D-gentiobiosyl)ester	*cis*-Crocin **4**	c-4GG	47.084	262, 326, 435, 457	976	C_44_H_64_O_24_
*cis*-crocetin (*β*-D-gentiobiosyl)(*β*-D-glucosyl) ester	*cis*-Crocin **3**	c-3Gg	48.252	258, 325, 435, 460	814	C_38_H_54_O_19_
*cis*-crocetin mono(*β*-D-gentiobiosyl) ester	*cis*-Crocin **2**	c-2G	49.511	262, 327, 433, 456	652	C_33_H_44_O_14_
*cis*-crocetin di(*β*-D-glucosyl) ester	cis-Crocin **2′**	c-2gg	49.711	262, 326, 434, 456sh	652	C_33_H_44_O_14_
*trans*-crocetin mono(*β*-D-glucosyl) ester	Crocin **1**	t-1g	50.592	258, 434, 459	490	C_26_H_34_O_9_
Crocin derivative	-	-	52.256	258, 434, 459	479~	C_44_H_64_O_24_
Crocin derivative	-	-	52.685	258, 327, 434	479~	C_44_H_64_O_24_
*cis*-crocetin (*tri*-β-D-glucosyl)(*β*-D-gentiobiosyl) ester	*cis*-Crocin **5**	c-5tG	53.893	263, 332sh, 445, 469	1138	C_50_H_74_O_29_
Crocin derivative	-	-	54.780	259, 323, 428, 452	479~	C_44_H_64_O_24_
Crocin derivative	-	-	55.140	245sh, 332, 431, 454	479~	C_44_H_64_O_24_
*cis*-crocetin mono(*β*-D-glucosyl) ester	*cis*-Crocin **1**	c-1g	55.614	259, 323, 428, 452	490	C_26_H_34_O_9_
Crocin derivative	-	-	57.965	258, 324sh, 434, 460	479~	C_44_H_64_O_24_
Crocin derivative	-	-	61.007	249, 326, 430, 453	479~	C_44_H_64_O_24_
*trans*-crocetin	-	*t*-crocetin	64.716	258, 434, 459	328	C_20_H_24_O_4_
*cis*-crocetin	-	c-crocetin	67.827	255, 318sh, 427, 453	328	C_20_H_24_O_4_

*—tentative detected according to [7,25,26,27]. **—Meaning of each letter in the abbreviation of the name of each crocetin ester: number (5, 4, 3, 2)—number of glucose molecules attached to the molecule of crocetin; t—triglucose; G—gentiobiose; g—glucose; n—neapolitanose.

**Table 5:** Content of identified secondary metabolites in *C. sativus* stigma (µg/g dry weight).

Code ^1^	Compound	Rt. min	Control	Vacuum3	CP3	EMF5
A	Picrocrocin	16.49	720.6 ± 88.4	965.6 ± 9.2 *	395.4 ± 2.4 *	847.3 ± 6.2 *
B	Rutin	20.81	889.0 ± 147.2	762.6 ± 21.0	171.0 ± 2.0 *	750.0 ± 33.1
C	*trans*-crocetin (*tri*-*β*-D-glucosyl)(*β*-D-gentiobiosyl) ester	33.19	2180.6 ± 19.4	2085.0 ± 130.1	591.2 ± 3.2 *	18,898.4 ± 17,021.5 **
D	*trans*-crocetin (*β*-D-neapolitanosyl)(*β*-D-gentiobiosyl) ester	34.93	867.2 ± 90.4	786.6 ± 6.5	76.4 ± 1.6 *	6356.2 ± 251.4 **
E	*trans*-crocetin (*β*-D-neapolitanosyl)(*β*-D-glucosyl) ester	35.63	357.0 ± 83.0	-	-	2034.7 ± 692.2 **
F	*trans*-crocetin di(*β*-D-gentiobiosyl)ester *	37.83	114,319.0 ± 513.6	124,350.4 ± 996.2	8949.1 ± 38.2 *	431,173.0 ± 965.0 *
G	*trans*-crocetin (*β*-D-gentiobiosyl)(*β*-D-glucosyl) ester	39.61	1714.2 ± 90.6	4225.0 ± 71.9 *	886.8 ± 9.2 *	37,187.7 ± 144.9 *
H	*trans*-crocetin mono(*β*-D-gentiobiosyl) ester	40.42	49,042.2 ± 1983.0	42,026.4 ± 251.0 *	6399.4 ± 101.8 *	188,232.6 ± 1415.9 **
I	*cis*-crocetin (*β*-D-neapolitanosyl)(*β*-D-gentiobiosyl) ester	42.12	1200.8 ± 24.6	575.0 ± 49.6 *	131.6 ± 14.8 *	781.9 ± 13.9 *
J	*trans*-crocetin di(*β*-D-glucosyl) ester	43.15	1735.6 ± 127.6	1363.8 ± 90.5 *	207.0 ± 1.4 *	1197.8 ± 34.7 *
K	*cis*-crocetin di(*β*-D-gentiobiosyl)ester	47.08	25,398.0 ± 195.0	12,675.6 ± 87.6	1899.8 ± 30.6 *	20,536.6 ± 74.9 *
L	*cis*-crocetin (*β*-D-gentiobiosyl)(*β*-D-glucosyl) ester	48.25	23,894.2 ± 137.8	21,464.2 ± 285.2 *	6270.6 ± 43.0 *	164,859.6 ± 14,739.5 **
M	*cis*-crocetin mono(β-D-gentiobiosyl) ester	49.51	3594.4 ± 162.4	1878.0 ± 19.0 *	192.2 ± 30.4 *	2530.7 ± 3.5 *
O	*cis*-crocetin di(*β*-D-glucosyl) ester	49.71	5529.8 ± 296.0	2812.1 ± 39.8 *	309.4 ± 19.2 *	17,151.2 ± 123.7 **
P	*trans*-crocetin mono(*β*-D-glucosyl) ester	50.59	-	257.9 ± 14.9 *	78.6 ± 5.8 *	1446.8 ± 124.9 *
Q	Crocin derivative	52.26	792.6 ± 56.6	400.5 ± 7.1 *	69.0 ± 0.18 *	4345.0 ± 401.2 **
R	Crocin derivative	52.69	-	-	61.6 ± 1.8	511.5 ± 33.4
S	*cis*-crocetin (*tri*-*β*-D-glucosyl)(*β*-D-gentiobiosyl) ester	53.89	251.4 ± 12.2	458.2 ± 11.8 *	95.6 ± 2.8 *	4876.2 ± 404.6 *
T	Crocin derivative	54.78	1289.6 ± 36.6	734.4 ± 3.0 *	171.8 ± 7.6 *	3238.4 ± 328.5 *
U	Crocin derivative	55.14	-	-	-	1531.6 ± 84.3
V	*cis*-crocetin mono(*β*-D-glucosyl) ester	55.61	1297.2 ± 16.0	737.6 ± 6.4 *	167.4 ± 9.2 *	3810.5 ± 157.4 **
W	Crocin derivative	57.97	1528.4 ± 85.6	1115.2 ± 55.7 *	144.6 ± 33.0 *	1374.1 ± 18.1 *
X	Crocin derivative	61.01	-	-	-	200.4 ± 23.6
Y	Safranal	62.28	8.4 ± 1.8	10.3 ± 0.5	1.60 ± 0.04 *	16.3 ± 0.2 **
Z	*trans*-crocetin	64.72	-	-	197.7 ± 1.2 *	225.4 ± 9.1 *
AA	*cis*-crocetin	67.83	-	-	-	204.6 ± 8.2

^1^—The code numbering of compounds in the chromatogram (Figure 6) and Table 5 is the same. Mean values ± SD are presented (n = 3). *, significantly different from the control group (*p* < 0.05); **, statistically significant difference compared to the control (*p* ≤ 0.001).

## Data Availability

Data are contained within the article.
